# Effects of Tungsten Disulphide Coating on Tapered Microfiber for Relative Humidity Sensing Applications

**DOI:** 10.3390/s21217132

**Published:** 2021-10-27

**Authors:** Norazida Ali, Saaidal Razalli Azzuhri, Md Ashadi Md Johari, Haroon Rashid, Muhammad Imran Mustafa Abdul Khudus, Mohd. Zulhakimi Ab. Razak, Zhe Chen, Norbahiah Misran, Norhana Arsad

**Affiliations:** 1 Department of Electrical, Electronic and System Engineering, Faculty of Engineering and Built Environment, Universiti Kebangsaan Malaysia (UKM), Bangi 43600, Malaysia; p96194@siswa.ukm.edu.my (N.A.); haroon@ukm.edu.my (H.R.); bahiah@ukm.edu.my (N.M.); 2 Department of Computer System and Technology, Faculty of Computer Science and IT, University of Malaya, Kuala Lumpur 50603, Malaysia; saaidal@um.edu.my; 3 Faculty of Engineering Technology, Universiti Teknikal Malaysia Melaka, Melaka 76100, Malaysia; ashadi@utem.edu.my; 4 Department of Physics, Faculty of Science, University of Malaya, Kuala Lumpur 50603, Malaysia; m.imran.mustafa@um.edu.my; 5 Institute of Microengineering and Nanoelectronics, Universiti Kebangsaan Malaysia (UKM), Bangi 43600, Malaysia; zul.hakimi@ukm.edu.my; 6 Department of Optoelectronic Engineering, Jinan University, Road Huangpu, District Tianhe, Guangzhou 510632, China; thzhechen@jnu.edu.cn

**Keywords:** tapered microfiber, humidity sensor, transition-metal dichalcogenide, tungsten disulphide (WS_2_)

## Abstract

Tungsten disulphide (WS_2_) is a two-dimensional transition-metal dichalcogenide material that can be used to improve the sensitivity of a variety of sensing applications. This study investigated the effect of WS_2_ coating on tapered region microfiber (MF) for relative humidity (RH) sensing applications. The flame brushing technique was used to taper the standard single-mode fiber (SMF) into three different waist diameter sizes of MF 2, 5, and 10 µm, respectively. The MFs were then coated with WS_2_ via a facile deposition method called the drop-casting technique. Since the MF had a strong evanescent field that allowed fast near-field interaction between the guided light and the environment, depositing WS_2_ onto the tapered region produced high humidity sensor sensitivity. The experiments were repeated three times to measure the average transmitted power, presenting repeatability and sensing stability. Each MF sample size was tested with varying humidity levels. Furthermore, the coated and non-coated MF performances were compared in the RH range of 45–90% RH at room temperature. It was found that the WS_2_ coating on 2 µm MF had a high sensitivity of 0.0861 dB/% RH with linearity over 99%. Thus, MF coated with WS_2_ encourages enhancement in the evanescent field effect in optical fiber humidity sensor applications.

## 1. Introduction

Silica fiber has received significant attention due to various applications in laser technology [1,2,3], sensor technology [4,5], plasmonic devices [6,7], telecommunications devices [8], and other applications. Moreover, due to recent technological development, silica fiber has experienced changes in structure, size, and composition, increasing its performance [9,10,11]. Hence, silica fiber has been used in critical areas for sensing purposes, such as the biomedical and chemical industries [12,13,14]. The size of silica fiber is the center of the discussion, as it can be manipulated and reduced to the micro-scale. This new development may increase the potential of silica fiber to be used widely and for numerous applications [15]. One techniques known as flame brushing can taper and reduce the silica fiber size down to the micro-region by heating and pulling using sufficient temperature flame [16,17]. The micro-silica fiber, better known as MF or tapered fiber, has a unique character due to its diameter and high index contrast between the core of the fiber and the air [18]. MF is the best choice in optical sensing applications based on the principle of the evanescent field, where strong interactions occur between the MF medium and its surroundings. This principle has been a significant advantage of MF, potentially being used in a limited area with high performances.

The MF structures used for relative humidity sensors can be classified into resonator and non-resonator types. A resonator is an advanced structure based on a closed-loop configuration that uses a large evanescent field created from self-coupling light and overlapping condition. This configuration enables the evanescent field to interact with the surrounding environment of the sensing region. As a result, the collected transmitted light is interpreted as the changed output power for sensing purposes. There are a few resonator-based structures, including the MF loop resonator (MLR) [19], the MF coil resonator (MCR) [20], the MF knot resonator (MKR) [21], and the whispering gallery mode resonator (WGMR) [22]. The working principle of the resonator-based humidity sensor is that the resonance wavelength is changed with the variation of humidity concentration. Besides sensitivity and linearity parameters, other sensing characteristics of resonators have been studied for the humidity sensors: intensity variation, wavelength shift, and Q-factor.

Unlike the resonator, the configuration of a non-resonator MF’s structure is straightforward and has been extensively used [23,24]. Besides, it has various forms, such as functional material coated sensors, Mach–Zehnder interferometer (MZI)-based sensors, micro-probes/nano-probes, grating-based sensors, and coupling-based sensors. For functional material coated sensors, the tapered region surface is coated with high refractive index material to create the functionality in humidity sensing to sustain the ability of waveguiding [25].

Two-dimensional (2D) transition metal dichalcogenides (TMDs) have recently piqued the interest of researchers due to their unique electrical, optical, chemical, and thermal properties [26,27,28]. Among TMDs, tungsten disulphide (WS_2_) has shown good potential for humidity sensing [29]. Numerous studies on humidity sensors that use 2D nanostructure-based materials coating as sensitivity catalysts have been reported recently [30,31,32,33].

In this work, the effects of WS_2_ coating on various waist diameters of MF using a facile and cost-effective method known as the drop-casting technique were explored for relative humidity sensing applications. Before that, using the flame brushing technique, we managed to form three sizes of tapered fiber region, i.e., 2, 5, and 10 µm. These MFs were then coated with WS_2_ and tested in humidity ranging from 45% to 90% for sensing purposes. The characterization of coated MFs was done by using a tunable laser source and optical power meter. The performances of coated MFs for the different tapered region in humidity sensing were evaluated and compared.

## 2. Fabrication and Preparation of Tapered Microfiber Sensor

**Figure 1:**
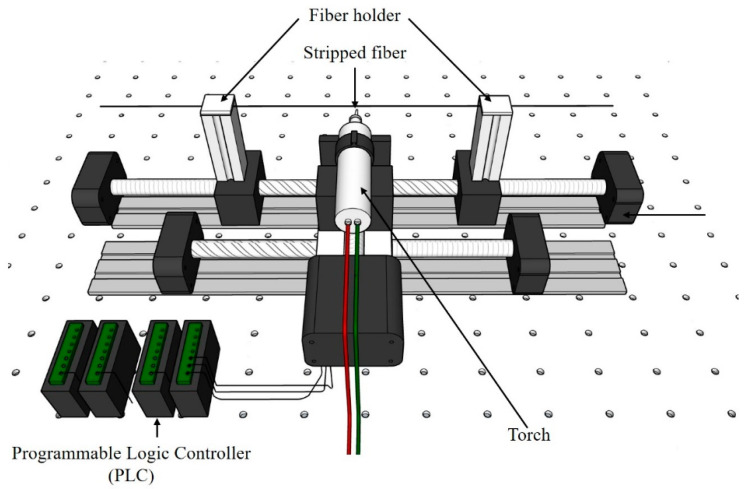
Schematic of the MF taper fabrication setup.

**Figure 2:**
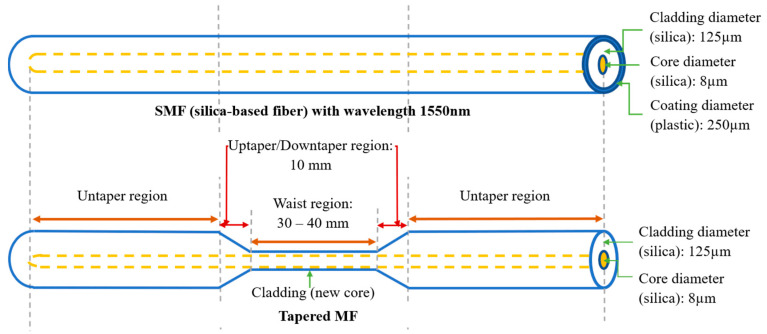
SMF before and after tapering process.

Figure 1 shows the schematic of the fabrication technique of the MF by means of a flame brushing method. The MF was fabricated from standard 125 μm SMF-28 using Programmable Logic Controller (PLC) stepper motor stages to pull the fiber while the flame from the torch moved to heat the stripped fiber. First, the outer plastic of the fiber was removed and the buffer was stripped, as shown in Figure 2. The fiber was then finely placed on the holder for the tapering process. The stripped area was heated gradually by manipulating the flame temperature, and the fiber was pulled from both sides to complete the process.

**Figure 3:**
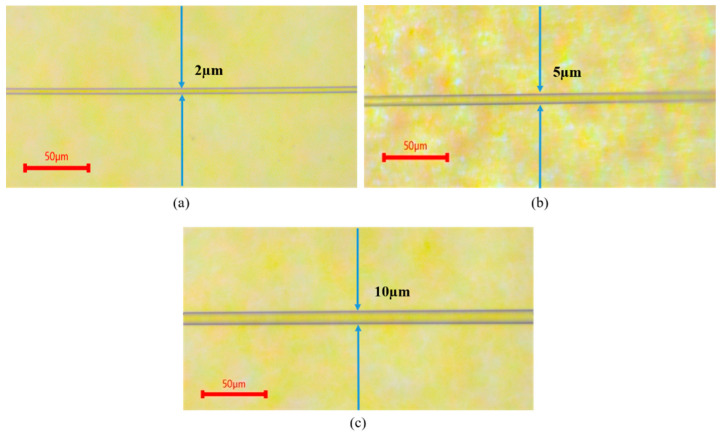
Microscopic image for tapered region MF with 2 µm (**a**), 5 µm (**b**), and 10 µm (**c**) diameter.

The MFs were then placed on glass slides and measured under a microscope to ensure the desired tapered region diameter depicted in Figure 3. After that, each MF sample was placed on a stage for the deposition process. The coating material, WS_2_ prepared in solution, was purchased from the Graphene Supermarket, which is practical for the SMF coating process. The MFs were coated at the tapered region with 10 μL of WS_2_ using the drop-casting technique and left in ambient environment for two hours to obtain a uniform layer formation on the tapered MF surface. The coated MFs were tested on a few humidity levels for sensing purposes.

**Figure 4:**
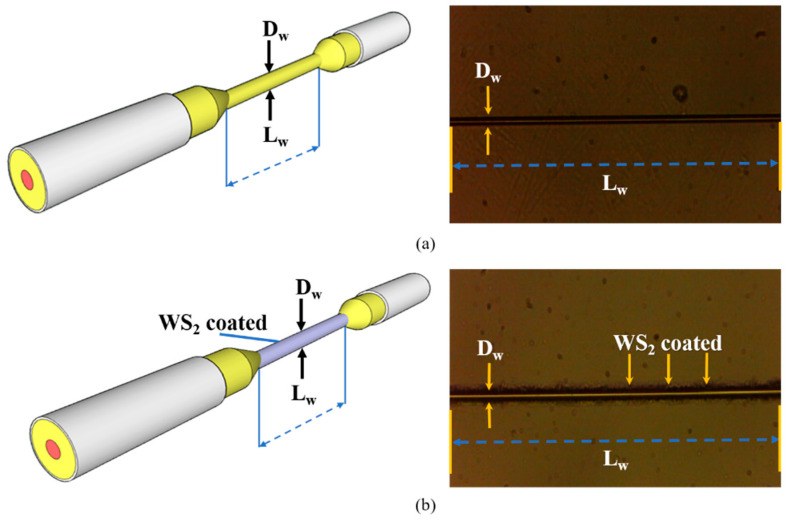
Microscopic images of MF structure (**a**) before coated and (**b**) after coated with WS_2_.

The size of the MF was determined by the time consumed by the heating process during the tapering procedure. The duration of tapering time is proportional to the size of the MF. Figure 4 demonstrates the characterization of MF in two parameters: waist diameter D_w_ and neck-to-neck length L_w_. The D_w_ is determined as the size of produced MF, which were 2, 5, and 10 µm with the waist-length range of about 30 to 40 mm.

## 3. Experimental Setup

**Figure 5:**
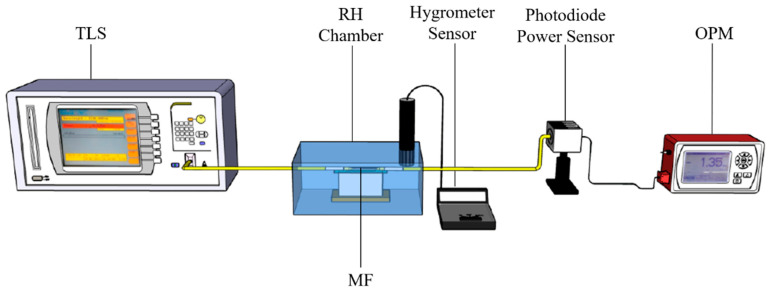
Experimental setup for MF RH sensing.

The performances of MF before and after WS_2_ coatings were compared. Figure 5 shows the experimental setup to investigate the performance of coated MF towards a different level of relative humidity percentage. Three differently coated MFs, i.e., 2, 5, and 10 µm, were placed inside the sealed chamber, whose ends were connected to the tunable laser source (TLS) and to the optical power meter (OPM). The chamber was also connected to a commercial hygrometer sensor (RS 1365) to monitor relative humidity levels. The experiment was performed at ambient temperature (25 °C) under an atmospheric pressure of 1.0 atm. The relative humidity was varied from 45% to 90% RH by using sodium hydroxide (NaOH) mixed with a drop of water inside the chamber.

The TLS (ANDO AQ4321D) at 1550 nm was used as an input, and the OPM (THORLABS S145C) was used to observe the output power simultaneously. Each coated MF of different sizes was repeatedly tested for three cycles to reduce the random errors during the experiment.

## 4. Sensing Mechanism

Despite tapered MF-based sensors having a simple form and being low cost, the detecting mechanism renders the RH sensor highly subject to light transmitted changes and fiber loss. The tapered MF sensing mechanism has relied on the variation of light intensity and transmission scheme, which can be used to evaluate the performance of the RH sensor. This sensor operates strongly dependently on MF that guides light through the tapered region (sensing area) and the interaction of evanescent field outside the fiber. It can be found that due to the strong evanescent field, the MF is extremely sensitive to the change in the surrounding environment, including temperature and RH.

For comparison, the actual RH level reading surrounding the tapered fiber inside the chamber was also measured using a hygrometer sensor as a reference. The changes in the surrounding environment caused by the presence of water vapor will result in the variation of the output transmitted power of the tapered MF, making it suitable to be used as a moisture sensor. The output transmitted power is identified by the modulation of light intensity according to the interaction between evanescent field absorption and the propagated light signal.

However, the tapered MF RH sensor found some difficulties in measuring the accurate value of sensing parameters since it is influenced by the fluctuations from the surrounding environment. Therefore, integrating the tapered MF with a coating material will improve the sensitivity of the RH sensor.

WS_2_ is among the TMDs that offers thermodynamic stability at room temperature, and that is capable of both absorption and desorption of water vapor. WS_2_ was deposited on the tapered MF as sensing material allowing for enhanced sensitivity of the RH sensor. When the sensor was exposed to water vapor, the tapered MF significantly interacted with the WS_2_, causing a change in fiber optical transmission at 1550 nm wavelength. As a result, even a tapered MF with a small waist diameter will achieve a high sensitivity RH sensor by enhancing evanescent field interaction between guided light and WS_2_ coating.

## 5. Results and Discussion

### 5.1. Characterization of Tapered Microfiber

**Figure 6:**
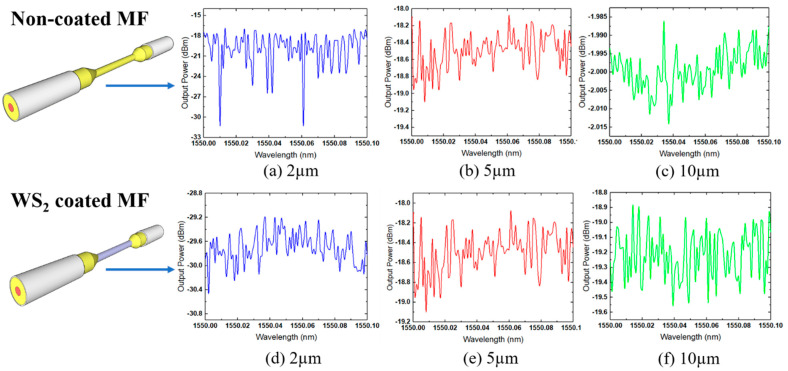
Non-coated MF transmission modes (**a**–*c*) and coated MF transmission modes (**d**–*f*) of 2, 5, and 10 µm.

The TLS was used to characterize the WS_2_-coated MF with a wavelength ranging from 1520 to 1620 nm. This experiment used the range of wavelengths from 1550 to 1550.1 nm with an interval of 0.001 nm. The OPM was used to collect the output power from the other end of the fiber. Figure 6 shows the transmission spectra produced by tapered MF with three different waist diameters (D_w_) and coated with WS_2_. The insertion loss from the 2 µm coated MF was −29.20 dBm, for the 5 µm MF it was −18.1 dBm, and for the 10 µm MF it was −18.9 dBm. From the results, 5 and 10 µm coating MFs had similar insertion loss values, whereas the insertion loss of the 2 µm MF was slightly lower. The size of non-coated MF affected the value of insertion loss. The coating diameter remained the same for all MFs: <10 µm.

The transmission spectra for the non-coated MF of three different sizes are depicted in Figure 6a. The insertion loss from the 2 µm non-coated MF was −17.00 dBm; it was −18.1 dBm for the 5 µm MF; and it was −1.985 dBm for the 10 µm MF. For 2 µm and 5 µm non-coated MFs, the insertion loss values were lower than 10 µm. The MF guides light as a single-mode waveguide, with the evanescent field guided outside the MF. This evanescent field is affected not only by the operating wavelength and the surrounding medium, but also by the diameter of the fiber [34]. Therefore, the size of the MF is still the main contributing factor for the insertion loss value, even for a non-coated MF. The waist diameter size of the MF sensor corresponds to the output transmitted power value. A small diameter of MF decreases the refractive index of the fiber and allows the light travelling through it to be refracted outside. Consequently, the insertion loss of output power will increase as the MF diameter decreases. For sensing applications, the smallest diameter is crucial to allow more light to be refracted over microfiber boundaries and become a sensing mechanism. The more light refracts, the more sensitive it becomes.

### 5.2. Structural, Morphological, and Compositional Analysis of WS_2_

**Figure 7:**
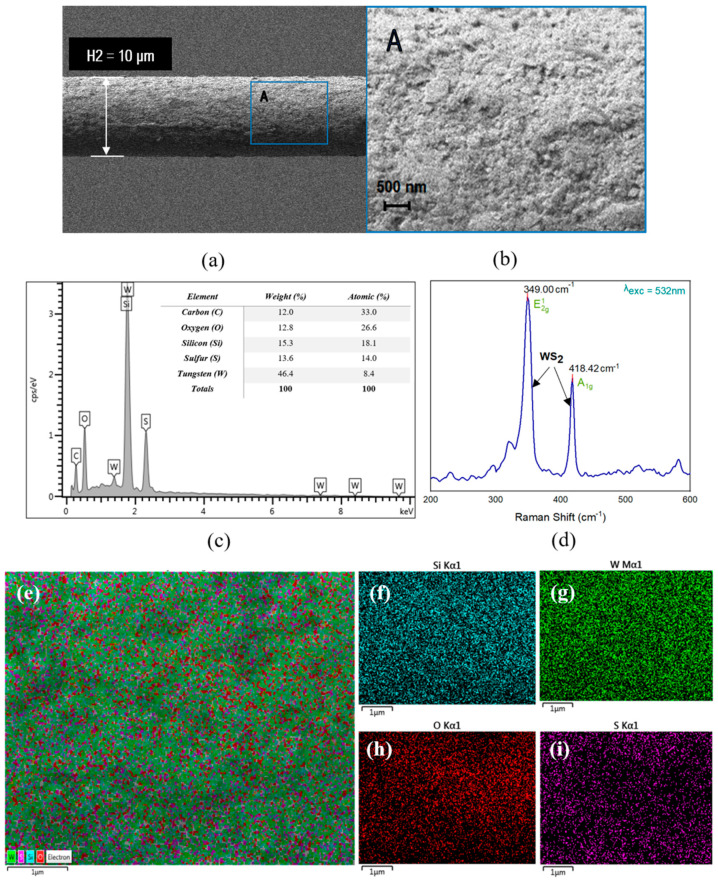
FESEM image of the MF coated with WS_2_ (**a**). An enlarged view of the region marked by a blue rectangle is MF coated with WS_2_ at 10,000× magnification (**b**). EDX elemental spectrum and quantification results for tapered MF coated with WS_2_ (**c**). Raman spectra of WS_2_ coated on MF by drop-casting (**d**). EDX-elemental mapping on the tapered region of MF coated with WS_2_ (**e**). Silicon, (**f**) tungsten (W) (**g**); oxygen (O) (**h**), and sulfur (**i**) distribution mapping.

The morphological characteristics of the MF were analyzed using field emission scanning electron microscopy (FESEM). Figure 7a represents the surface morphology of WS_2_ coated (thin layer) on top of the MF waist region. The enlarged image of the WS_2_ can be seen in Figure 7b, which adhered to the MF surface with a diameter range of 400 to 600 nm. The elemental compositional analysis and elemental distribution mapping were performed using energy-dispersive X-ray spectroscopy (EDX). Figure 7c represents EDX analysis of the 10 µm WS_2_-coated MF. The inset of Figure 7c tabulates the percentual weights and atomic percentages of the elemental compositions of materials. The presence of tungsten (W) and sulfur (S) confirms the successful deposition of sensitive sensing material (WS_2_). Silicon (Si), carbon (C) and oxygen (O) peaks arose from MF. The normalized S/W ratio was calculated to be 1.667, which is evidence of non-stoichiometric WS_2_ presence. The deficiency of S atoms is attributed to the presence of O atoms from the environmental during the deposition process performed at room temperature. Figure 7f–i represents the elemental distribution mapping of Si, W, O and S, respectively.

The structural properties of coated MF were obtained using Raman scattering analysis within the spectrum range from 330 to 450 cm^−1^. Figure 7d divulges the bonding structure of the thin WS_2_ layer. Two dominant peaks are shown within the spectrum range. Both originated from an in-plane optical mode known as phonon mode (E^1^_2g_) and from an out-plane vibration mode caused by S atoms, confirming the successful growth of WS_2_ on the MF surface. The lattice vibration is caused by the weak van der Waals interlayer forces. Both E^1^_2g_ and A^1^_g_ modes are apparent at 349 and 418.42 cm^−1^, respectively. The separation (Δ) between two peaks (E^1^_2g_ and A^1^_g_) was calculated as 69.42 cm^−1^, which validates the presence of WS_2_ [35].

### 5.3. The Performance of the RH Sensor

**Figure 8:**
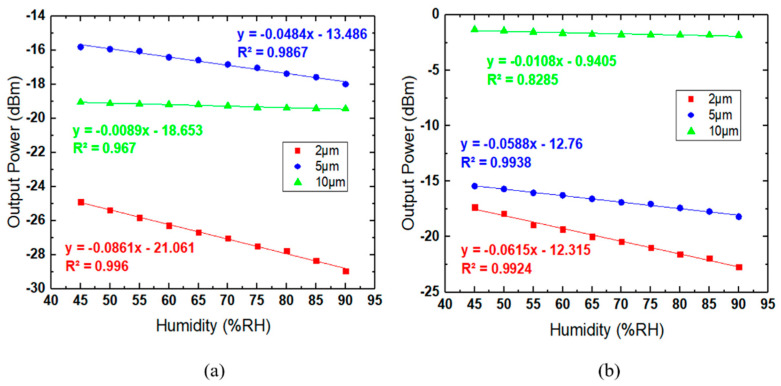
Transmitted power value for three different diameters of WS_2_: (**a**) coated MFs and (**b**) non-coated MFs. Power varies with the percentage of humidity.

Sensitivity and linearity values for three different sizes of coated and uncoated MFs are shown in Figure 8. Sensitivity is defined as the change in output power for each RH level [36]. Linearity is the interaction of output powers concerning each percentage of RH [37]. Figure 8a shows the sensitivity and linearity results, among which 2 µm coated MF recorded the highest values: 0.0861 dB/% RH for sensitivity and 99.8% for linearity. Figure 8b represents the sensitivity and linearity values for non-coated MF. The 2 µm non-coated MF had the highest performance parameters with a sensitivity of 0.0615 dB/% RH and linearity of 99.6%. The tapered regions of 5 µm and 10 µm show lower sensitivity and linearity than the 2 µm tapered MF. The results for all parameters are tabulated in Table 1. In both graphs, the 2 µm coated MF shows the best performance for sensitivity and linearity. It is also shown to have more sensitivity towards a different level of humidity. Moreover, by coating the MF with WS_2_, the humidity sensor recorded higher sensitivity and linearity than the non-coated MF. These experiments show that WS_2_ has the potential to improve humidity sensing capabilities.

**Figure 9:**
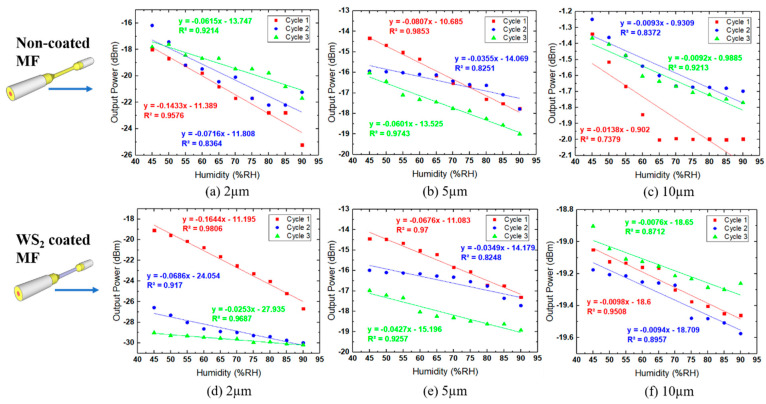
Non-coated MF (**a**–**c**) and coated MF (**d**–**f**) of 2, 5, and 10 µm repeatability results with different levels of humidity.

Figure 9 represents the repeatability results for coated and non-coated MF on all three diameter sizes. This process is vital to reduce random error in measurement, which affects the sensing process. For every MF condition, it was repeated three times with the same setup. The sensitivity and linearity were calculated to ensure that all data collection was always on track. The results show that the 2 µm MF exhibited the best sensitivity toward RH changes for both coated and non-coated MFs, and among all the MF, the 2 µm coated MF had the optimum RH sensing performance.

## 6. Conclusions

In this paper, we demonstrated the performance of tungsten disulphide (WS_2_) as a coating material on tapered MF for RH sensing applications. We used it to enhance sensitivity with changing RH. The MF was formed with three different waist diameter sizes, i.e., 2, 5, and 10 µm, using a flame brushing technique developed in-house. The fabricated MFs were then prepared in two sets, non-coated and MFs coated with WS_2_; each set had a similar size of tapered region. Both sets were experimentally characterized using TLS with wavelength ranging from 1550 to 1550.1 nm with a step interval of 0.001 nm. For these experiments with 45–90% RH, the results were compared and evaluated. The sensitivity and linearity parameters were analyzed to identify the sensing performance. The optimum performance was recorded for the WS_2_-coated 2 µm MF with sensitivity and linearity values of 0.0861 dB/% RH and 99.8%, respectively. These results show the potential of WS_2_ to become the potential sensing material for optical sensing applications.

## Figures and Tables

**Table 1:** Analysis of different diameters of MF with WS_2_ performance in humidity sensing activity.

Parameters	2 µm		5 µm		10 µm	
	Non-Coated MF	Coated MF	Non-Coated MF	Coated MF	Non-Coated MF	Coated MF
Linearity (%)	99.6	99.8	99.3	99.3	91.0	98.3
Sensitivity (dB/% RH)	0.0615	0.0861	0.0484	0.0484	0.0108	0.0089
Linear Range (% RH)	45–90	45–90	45–90	45–90	45–90	45–90

## Data Availability

Not applicable.
